# Unfulfilled expectations to services offered at primary health care facilities: Experiences of caretakers of underfive children in rural Tanzania

**DOI:** 10.1186/1472-6963-12-158

**Published:** 2012-06-14

**Authors:** Catherine Kahabuka, Karen Marie Moland, Gunnar Kvåle, Sven Gudmund Hinderaker

**Affiliations:** 1 Centre for International Health, Faculty of Medicine and Dentistry, University of Bergen, Bergen, Norway; 2 Department of Nursing, Bergen University College, Bergen, Norway

## Abstract

**Background:**

There is growing evidence that patients frequently bypass primary health care (PHC) facilities in favour of higher level hospitals regardless of substantial additional time and costs. Among the reasons given for bypassing are poor services (including lack of drugs and diagnostic facilities) and lack of trust in health workers. The World Health Report 2008 “PHC now more than ever” pointed to the importance of organizing health services around people’s needs and expectations as one of the four main issues of PHC reforms. There is limited documentation of user’s expectations to services offered at PHC facilities. The current study is a community extension of a hospital-based survey that showed a high bypassing frequency of PHC facilities among caretakers seeking care for their underfive children at two district hospitals. We aimed to explore caretakers’ perceptions and expectations to services offered at PHC facilities in their area with reference to their experiences seeking care at such facilities.

**Methods:**

We conducted four community-based focus group discussions (FGD’s) with 47 caretakers of underfive children in Muheza district of Tanga region, Tanzania in October 2009.

**Results:**

Lack of clinical examinations and laboratory tests, combined with shortage of drugs and health workers, were common experiences. Across all the focus group discussions, unpleasant health workers’ behaviors, lack of urgency and unnecessary delays were major complaints. In some places, unauthorized fees reduced access to services.

**Conclusion:**

The study revealed significant disappointments among caretakers with regard to the quality of services offered at PHC facilities in their areas, with implications for their utilization and proper functioning of the referral system. Practices regarding partial drugs administrations, skipping of injections, unofficial payments and consultations by unskilled health care providers need urgent action. There is also a need for proper accountability mechanisms to govern appropriate allocation and monitoring of health care resources and services in Tanzania.

## Background

To facilitate access to health care, many low-income countries have delegated care for uncomplicated health problems to primary health care (PHC) facilities. In Tanzania, these are termed dispensaries or health centres. According to Tanzania health policy 2003, dispensaries are designed to offer outpatient services including reproductive and child health services, and basic diagnostic services [1]. Health centres offer outpatient and inpatient services, maternity care, laboratory, dispensing and mortuary services [1]. Dispensaries in Tanzania are usually run by non-physician clinicians called clinical officers, who are trained to attend simple health conditions and refer complicated ones to hospitals. These are assisted by one or two nurses. Health centers are staffed by a wider range of more qualified health workers. If effectively utilized by the targeted population, these facilities serve to reduce overcrowding at higher level hospitals that are designed to manage more complicated cases through the provision of more specialized care.

It is well known that providing quality care to sick children particularly at an early disease stage is crucial for attaining better health outcomes including preventing child deaths from treatable illnesses. However, the quality of care provided at most PHC facilities in developing countries continues to be questionable [2,3]. Problems of frequent shortages of drugs, diagnostic equipments and health workers characterize most of such facilities [2,3]. As much as 72% of Tanzania’s population live within 5 km and 93% within 10 km of a health care facility [4]. With this density of PHC facilities, access in terms of distance is reasonable but the problems of care-seekers bypassing these facilities have repeatedly been reported [5-7]. In a population-based survey, in rural western Tanzania, it was reported that 44% of women seeking institutional delivery had bypassed their nearest PHC facility [5]. In this study, the women reported that quality of care (e.g. best provider, availability of drugs) and trust in health workers were the main reasons for selecting the health facility for delivery. Results from a household based survey in Lushoto district showed that patients bypassed their lower level of care to seek hospital treatment because of poor quality of services and poor availability of drugs [6]. The problems of bypassing lower level facilities have also been reported in Kenya, Sri-Lanka and Namibia [8-10]. Reasons given for bypassing PHC facilities in all the above mentioned studies can be summarized as “unfulfilled expectations”, referring to dissatisfaction and disappointment from the quality of the services offered at such facilities.

Organizing health services around people’s needs and expectations was strongly emphasized in the World Health Report of 2008 as one of the major four PHC reforms termed “services delivery reforms”[11]. The report brought into attention the fact that the PHC movement had underestimated the speed with which the transition in demand for professional care would bypass the initial attempts to expand rapidly access to health care by relying on sub-optimal care provided at most of the PHC facilities. The report further claimed that, “neglecting people’s needs and expectations is a recipe for disconnecting health services from the communities they serve”.

There is limited documentation of the user’s expectations to services offered at PHC facilities. The current study is a community extension of a hospital-based survey that showed a high bypassing frequency of PHC facilities among caretakers seeking care for their underfive children at two district hospitals in the study area [12]. Our study objective was to explore caretakers’ perceptions and expectations from the quality of services offered at PHC facilities in their area with reference to their experiences at such facilities.

## Methods

### Study context

The study was carried out in Muheza district of Tanga region in north-eastern Tanzania. Muheza district is located about 25 km from the city of Tanga. Based on the 2002 census and an annual population growth rate of 1.4%, the district had a projected population of 306,862 in 2009 [13]. This population is served by 59 dispensaries (49 public, 10 private), six health centres (4 public, 2 private), and one district hospital [14]. The district is predominantly rural, and the people are mainly subsistence farmers of maize, cassava, oranges, coconut, rice and banana. Malaria is the leading cause of admissions and deaths among under-five children [14,15]. In 2005, the underfive mortality rate in Muheza district was 150/1000 live births (140/1000 live births in the whole region of Tanga). The above features make the region as well as the district similar to most of the rural Tanzania [16].

### Study design and data collection

Due to its exploratory nature, the study employed qualitative research methods. We conducted four focus group discussions (FGDs) in four villages of Muheza district in September 2009 (Table 1). The villages were purposely selected from a list of eleven villages where the hospital survey participants reported to live. We used three main criteria for villages selection; 1) A village must be at least 10 km from the district hospital, 2) It must have a dispensary or health centre nearer than the district hospital and 3) It should have a significant number of bypassers (caretakers who did not at all utilize their nearer dispensary or health centre during the index child’s sickness) and non-bypassers (caretakers who utilized their nearer dispensary or health centre prior to coming to the district hospital) as documented in the hospital survey [12]. In addition to above, we also selected villages in different geographical directions from the district hospital.

**Table 1:** Composition of FGDs

**FGD number**	**Participants**	
	**Men**	**Women**
Village 1	0	9
Village 2	7	5
Village 3	4	10
Village 4	0	12

FGD participants were recruited one week before the meeting, through a village leader who was informed of the criteria for the selection of the study participants. The inclusion criteria were that the participants 1) should have at least one child less than five years under their care and 2) there should be only one caretaker participating per household. We advised the village leader to pick households skipping several in between even if they had a potential participant so as to have a wider area represented. A total of 47 women and men participated in four focus groups, each comprising of 9–14 participants. The majority were women reflecting the fact that women are the ones who commonly take children to the hospital within the study setting. Two FGDs were mixed while two were with women only. The discussions were held either in village government halls or a school classroom.

A guide was used to introduce topics for discussion in the form of questions. We left caretakers to discuss among themselves with the principal investigator as a moderator. Topics discussed included available health care options, quality of available primary health care services, barriers and obstacles faced by caretakers while seeking care for their sick underfive children at the PHC facilities in their areas, and reasons for bypassing PHC facilities. We added emerging new issues in the guide as the discussions progressed and we asked them in subsequent FGDs. The discussions were conducted in Swahili (the national language) by the principal investigator as a facilitator and two research assistants and were all tape-recorded. The discussions lasted between 55 and 90 minutes. At the end, participants were given an opportunity to ask general questions on health issues, and the principal investigator and one research assistant who was a clinician responded accordingly.

### Ethical consideration

We obtained verbal consent for participation and audio taping from all the study participants prior to commencement of the discussions and after having explained the aim of the study. To secure confidentiality and facilitate an open discussion, we did not ask for a written consent and did not record the names of the participants. Only their sex was recorded. The study was approved by the Medical Research Coordinating Committee at the National Institute for Medical Research in Tanzania.

### Data analysis

Data was manually analysed using the principles of thematic content analysis [17]. Audio materials were transcribed and analysed in the original discussions language, Swahili. Analysis commenced by examining the data material thoroughly so as to identify units in the text that were relevant to the subject in question. The identified units were then coded, categorized and later grouped under meaningful themes. Initial word to word transcription of the audio materials was done by one of the research assistants. Translation into English was done by the principal investigator at a later stage when meaningful themes had been identified. The second author took part in the analysis.

## Results

### Unfulfilled expectations

The findings reveal that caretakers had certain expectations to the quality of care to be offered when they brought their children to a health facility. When these expectations were not met, caretakers became dissatisfied. The study established substantial frustrations among caretakers related to availability, accessibility and acceptability of the services offered by PHC facilities in their area. Experiences not only of lack of quality services but also unacceptable health workers’ behaviours dominated the discussions. In the following section, we present the findings as caretakers’ unfulfilled expectations to the health services provided at the primary care level.

### Expecting examinations before treatment

#### No tools, no touch

Our study established a growing demand among caretakers wanting to know for sure what’s wrong with their children before they are given treatments. Caretakers expressed their disappointments regarding the common practice among clinicians of prescribing drugs to their children without having any investigation performed. This was also given as the main reason for bypassing PHC facilities, as one female caretaker expressed: “Nowadays people are civilized, they are afraid of giving the child drugs before she is investigated. They want to be sure if it’s real malaria. The fever could be just a result of the teething process. So they go there (district hospital) to get the child investigated”. (Woman, Village 2).

Although health centres and even dispensaries are supposed to provide basic diagnostic services, such services were reported to be almost never available. Caretakers in this study expressed their high demand of such services: “The main thing that bothers us and makes us go to town is the lack of diagnostic services. They should at least bring diagnostic services to our dispensaries. Because we know even if you go there (dispensary), the child will just be given ALU (first line anti-malarial drug) but may be the sickness is not even for ALU.”(Woman, Village 2).

In one village, caretakers claimed to previously be provided with diagnostic services at their facility. However, the laboratory is no longer running because of the frequent shortage of laboratory reagents. She explained: “When they first brought the diagnostic services, people were being investigated normally for malaria, fever etc., but now they tell you that there is no reagent. Even temperature is not taken. They will just touch the child, and if the body is warm then you are told she has malaria. You will just be given drugs to give the child at home”.(Woman, Village 3).

In addition to not receiving diagnostic services caretakers also reported that even basic clinical examinations were sometime not performed by the attending clinician. Commonly, the attending clinicians asked questions and prescribed drugs without even touching the child as expressed in the quote below: “There used to be that thing they put in your mouth or armpit, for checking temperature. Now even temperature is not taken. They do not even touch the child, you talk they write” (Woman, Village 3).

#### Medical tools as table decorations

Caretakers claimed that, even when the tools for clinical examination were available, they were often not utilized by the attending clinicians. Sometimes they were just displayed/left on the table as the following quote illustrates: “Another thing is that thing they wear around the neck, for examining the chest and the heart (stethoscope). For them, it is a table decoration. Every time you go with your child you find it stuck on the table. He just writes, he does not even look at your face. They should take it back to Teule (district hospital), if they do not know its use” (Woman, Village 3).

### Expecting drugs to be available and sufficient

Frequent shortage of drugs was reported to be commonly encountered at PHC facilities. Caretakers reported often to be provided with the antimalarial ALU® (which was commonly available) and ordered to buy other drugs. Occasionally, even this malaria drug was not available at the facility nor in drug shops, in which case caretakers were forced to buy other types of malaria drugs, like Fansidar® which is no longer recommended by the Ministry of Health and Welfare (MoHW). One caretaker reported: “Antimalarial drugs are as well not available sometimes. We are used to go and buy these old ones like fansider. The drug will help the child for three, four days, and then malaria comes back very severe” (Woman, Village 1).

#### Unsafe drug dispensing practices

In connection with drug shortages, an alarming drug dispensing practice was reported in two villages where FGDs were conducted. In one of the villages, a woman said: “They ask if you brought a bottle, if you do not have they tell you to go and buy. They pour you some amoxicillin and you go. One bottle is divided among two to three children” (Woman, Village 3).

A caretaker in another village reported the same experience: “You may reach there and be told to go and bring a container. The mother is supposed to go back home and bring a container so that they pour you some drug. They measure mills and distribute one bottle among two to three children” (Woman, Village 1).

Caretakers raised several concerns pertaining to the above practice. First, they complained that most of the time the drug provided was not sufficient for the number of days prescribed: “You are told to give the child drug for five days, after three days the drug finishes and you do not know what to do” (Woman, Village 3).

Secondly, caretakers expressed their worry about the hygiene of the containers they used for taking the drug, since most of them were obtained under emergency conditions: “You are told to go look for a container at home or shops. You do not know of the safety of that container where you obtained it. They pour you drug, you give the child and diarrhoea becomes worse” (Woman, Village 1).

#### Health worker dependent drug availability

In one of the villages caretakers reported a sudden change pertaining to the availability of drugs after having new clinicians at their facility. One male caretaker reported: “Before they could bring drugs, but after five or ten days you are told drugs are out of stock. But since they brought these ladies, we are thankful because you could go even the whole month and drugs are available. It takes a long time before you are told drugs are out of stock” (Man, Village2).

### Expecting health workers to be available

Unreliable availability of health workers at PHC facilities was another issue raised across all the four FGDs. The number of staff at most facilities was claimed to be insufficient to provide the expected services, and this was even more aggravated by their frequent absenteeism. Commonly only one nurse was available who had to do everything, as the quotes below points out: “Sometimes you find that the main doctor is not around and only one nurse is doing everything. She then will become the doctor. She will be the one taking care of the MCH clinic services, she will be the one working at the Antenatal clinic etc.” (Woman, Village 4).

The same concern was raised by caretakers from a different village as reported by this woman: “Sometimes there is only one nurse, when others have gone on holiday. The same nurse has to give drugs to patients, attend children at MCH, and take care of pregnant women. By the end of the day she is very tired. She can’t do her job properly.” (Woman, Village 1).

### Expecting facilities to be open

Dispensaries in Tanzania operate between 8 am and 4 pm and are closed on weekends and public holidays. However, it was reported that sometimes the facilities were closed even during the week days when no health staff was available to provide services. Ideally when the dispensary is closed, the health staffs who are supposed to reside within the same community can still be contacted at their homes and are expected to provide services if the need arises. However, staffs are usually inaccessible after opening hours since most of them do not live within the communities they serve. One woman complained: “If your child gets sick at night, there is nowhere to run to until morning. If you have money then you will look for transport and take her to Teule (district hospital) the same night. If you can’t afford then your child might die while you are watching her (Woman, Village 3).

#### Skipped injections due to facilities closure

If a child was prescribed hourly injections and the dosage fell on weekends, caretakers reported that the injections were skipped until the next working day: “On weekends the situation is even worse. Lets say today is Friday and your child has been prescribed hourly injections. When it reaches Friday evening, all nurses leave, as most of them live in Muheza (district centre). So the dose will be stopped until Monday when they come again. There is no service on weekends” (Woman, Village 1).

#### Wellbeing of newborn babies

The problem of unavailability of services in the evenings, weekends and holidays, was also reported as being of major concern for pregnant women and the outcome of the newborns in the area. It was reported that it was always a big challenge when a woman went into labour when the facility was closed, and most of the time there was no health staff available to assist with the delivery process as they did not reside within the community. One woman expressed: “… There is no nurse here to deliver the child at night. If labour starts at night you have to look for transport to go and deliver at Teule hospital, even if its 2 am. Nurses are only available during the day, when it reaches 4 o’clock they close and leave until the next day. That’s why we are always full at Teule (District hospital). When you start feeling bad you decide to go straight to Teule as early as possible (Woman, Village 4).

In one village, a male village leader reported assisting pregnant women in labour at night several times, either to look for transport to take them to the district hospital or to mobilize funds from neighbours to pay for the transport costs. He also claimed that there were several occasions when there was no enough time to look for transport before the baby came out, in which case, himself and his wife had to assist with the delivering process. Below are his words: “I must tell you, I have delivered women, not once. I am not a nurse but sometimes a woman is brought to me and she is ready to deliver. It’s not possible to put her in the car and there is no nurse to assist, so I have to do it. In this case I don’t know whose fault to say it is” (Man, Village 2).

#### Unavailability of electricity as a reason for facilities closure at night

Even when the health worker was willing to work at night, the working environment was reported not to be supportive. Caretakers in one of the villages reported that even when they got assistance from the nurse who lived within the village, they were supposed to bring a lamp to the dispensary since there was no electricity or any other alternative source of light. One caretaker reported: “When you knock at the nurse’s door, before she even comes out she asks if you brought a lamp. If you don’t have she tells you she can’t work because the dispensary does not have light. That has happened to me three times. But because I have relatives closeby I go to them and they give me a lamp. Sometimes they tell me it has no kerosene, so I have to look for kerosene elsewhere” (Woman, Village 3).

In one village, a village leader reported that nurses in his area were being paid night allowances, and he was even requested to monitor their presence at night. However, the allowances were discontinued, and nurses are no longer working at night.

### Expecting to be treated well

Undesirable health workers behaviours was another main complain raised by caretakers across all FGDs. Caretakers expressed their concerns on the way nurses and sometimes doctors treated them. Below are some of the behaviours that were of concern:

#### Unnecessary delays

Unnecessary delays particularly among the nurses came as one of the main complaints. Caretakers reported leaving their homes early so as to get to the facility early but most of the time they were left waiting in queues for long periods without any good reason as one caretaker expressed: “You might reach there and find two nurses, one cleaning and other arranging files. You went so early, lets say you arrived a few minutes before 8 am, but you may not be touched or even spoken to until 11 am” (Woman, Village 3).

Another woman from the same village added: “They also have a habit of going for tea like students. You may be told the doctor has gone for tea and you may wait for 3 hours and he may not be back. Or they may just be talking inside and you are waiting outside. That’s why it is always full at Teule because of nurses’ ignorance here” (Woman, Village 3).

#### Lack of urgency

It was reported that even when caretakers requested for immediate attention due to the seriousness of their children’s condition, still lack of urgency dominated. Caretakers reported sometimes to receive impolite answers from the nurses and doctors when they approached them to request for urgent care. The quotes below illustrate: “When you approach the nurse and tell her that my child’s condition is not good she tells you ‘do not teach me my job’. Then you remain with nothing to do but wait while observing your child until when she feels like helping your child by herself.” (Woman, Village 3).

Another woman in a different village added: “Sometimes when you see that your child’s condition is not good and approach the nurse she may chase you away and even yell at you ‘go sit and wait outside’. (Woman, Village 4).

A man from the third village reported his experience, related to the above complains, at one primary health care facility in his area: “I guess two years ago, while we were on our way to the facility, my child got very high fever. While at the facility waiting for our turn to see the doctor, my child started convulsing. I requested other patients ahead of me so that I go in first, and due to my child’s obvious condition they allowed me. However, soon after I entered the doctor’s room he yelled at me “get out, who told you to come in”. I told him to look at my child’s condition and that my child was convulsing but he yelled at me “go away, do you know what a convulsion is?” I went back outside with my convulsing child. Fortunately a nurse was passing by and got to see my child’s condition and took him to the doctor and that’s when he got to attend him” (Man, Village 2).

#### Lack of sympathy

Lack of compassion by health workers to sick children was among other complains raised by caretakers. The latter story is a good example of this. It was as well reported that clinicians referred to by caretakers as doctors, commonly did not even touch the child. This was a big disappointment to most caretakers who expected their children to be properly examined. One caretaker expressed: “Your child’s name might be called when it is your turn to see the doctor but when you get to sit near the doctor he may tell you to move away and that your child smells urine, claiming that we don’t clean them properly. But you tell me, from the time I left my house, all the way to the hospital, if my child doesn’t pass urine then wouldn’t that be a sickness? And when you tell him the child’s problem he just writes without even touching the child because he smells bad” (Woman, Village 3).

A man from a different village had these to say: “I will just give an example; there was a doctor here who has been recently transferred. No matter how serious your child was, when you requested for help outside working hours, he would just tell you, while sitting on his house veranda, this is neither a hospital nor working hours. But thank God that we now have very kind women who are ready to assists us any time” (Man, Village 2).

### Expecting services without payments

According to Tanzanian health policy, health care services to children below five years is supposed to be provided free of charge at public health facilities. However, caretakers claimed to incurr some costs for various services when they brought their children to the primary care facilities. This was reported in three villages while in one village caretakers reported not to pay at all. The payments were reported to be for various services and were differed from one facility to another. Payments could be for consultation, drugs, investigations (when available) and facility guard. The quotes below illustrate: “To see a doctor is two hundred, the drug that you are poured in a container costs two hundred and five hundred for malaria investigation, total nine hundred. There at Mkanyageni children are not free” (Woman, Village 4).

Another woman from the same village added: “Here at our facility the only investigation available is that for malaria, which costs five hundred. If you don’t have five hundred they will just give you ALU (first line antimalarial drugs) to give the child at home. We also pay one hundred for the guard” (Woman, Village 4).

In one village, caretakers reported to pay for the “free-of-charge maternal and child health (MCH) services”, like monthly child weighing. The quote below illustrates: “Consultation and treatment costs one thousand and we pay two hundred for the guard who watches the dispensary. Weighing a child at the clinic costs two hundred” (Woman, Village 1).

However, Village 1 was the only village where caretakers reported paying for monthly child weighing. Other villages claimed not to pay for this service. We probed further to find out what happened when caretakers were not able to pay for these services and below was a response from one of the villages: “I f you don’t have two hundred they will not weigh your child and if you don’t have one thousand you won’t get any service” (Woman, Village 1).

Inaccessibility of services resulting from the inability to pay was also mentioned in the quote above by a woman from Village 4, who claimed that even though malaria investigation was available at their nearer PHC facility children were just prescribed ALU if caretakers were not able to pay for them. Occasionally, caretakers reported to pay and still be told that the drugs were out of stock and given a prescription to go and buy them elsewhere. Caretakers complained that this caused a lot of inconveniences as they had to look for other funds to buy the drugs which could take sometime while the child continues to suffer.

### Expecting written referral information

Another thing complained about by caretakers was the lack of proper referral services when their children were referred to a higher level hospital. Across all the focus group discussions, caretakers complained of the fact that they were only given verbal referrals, and no written document was provided for them to take to the referred hospital. Caretaker’s main concern was that they were not able to answer correctly some of the questions that were asked at the referred facility. They expressed their wish to have some sort of a written report from the primary care facility which would assist them. The quotes below illustrate: “He just tells you to take the child to Teule. Its only words while he is the one who treated the child without improvement. Why not give me any introduction so that when I reach there they know where I started. When I arrive at Teule (district hospital) I have to start afresh while his introduction would have helped.” (Man, Village 2).

Another caretaker from a different village had this to say: “No arrangements, its just verbal, ‘take a child to Teule’, finish. Whether you went or not they don’t care. You won’t even be given a nurse to accompany you or even a piece of paper to say this child we failed, help her there, its just words. Also if you ask questions you may be answered rudely.” (Woman, Village 3).

## Discussion

Concerns raised by caretakers in this study, related to their unfulfilled expectations from services provided at PHC facilities, provide an insight into barriers to services utilization other than geographical accessibility. The findings provide relevant information on outcome indicators of access i.e. utilization and satisfaction, by the targeted population [18]. Obrist et al. defined access to health services as five dimensions namely availability, accessibility, affordability, adequacy and acceptability [19]. He claimed that, these dimensions directly influence the course of the health seeking process and hence utilization of the services. We argue that the same dimensions define quality of care as perceived by users of a given facility. Hence, in the following section we discuss our findings based on the above five dimensions of access.

### Availability and adequacy of services

The current study was conducted among caretakers who lived within an area with a good geographical access to a PHC facility (dispensary and/or health centre); however, the facilities were reported to be characterized by frequent shortages of staff and equipments (including essential medicines and diagnostic services).

The discussions established a growing demand among caretakers regarding the provision of diagnostic services at PHC facilities in their area. This was found to be the most common reason for bypassing PHC facilities also in the study at the two district hospitals in the area [12]. This could be explained by the raising awareness among caretakers regarding standard childhood infection management, which requires confirmation of diagnosis through diagnostic tests. It could also simply be a result of caretakers’ previous experiences of treatment failures, when their children were provided drugs at such facilities without having any tests performed. Some diseases like pneumonia and diarrhoea can be correctly diagnosed by thorough history taking and clinical examination. Instruments like IMCI and WHO guidelines for the management of common illnesses with limited resources have been proven successful in achieving this goal [20,21]. However, for a disease like malaria a confirmatory test is very crucial in ascertaining the correct diagnosis. Most PHC facilities in Tanzania lack diagnostic equipments and it is a common practise for clinicians to rely solely on history and clinical findings to make a diagnosis [22]. As a malaria endemic area, many clinicians in Tanzania prescribe antimalarials to children presenting with a history of fever, often without a thorough clinical examination to rule out other infections (sometimes even when tools are available to enable them to do so). Many of these children receive no additional antibiotics. Malaria prevalence has been going down in Tanzania as well as in other parts of sub-Saharan Africa [23], and this may indicate that if not properly investigated children with fever may wrongly be diagnosed as malaria cases and the treatment provided may not necessarily lead to recovery.

Findings from a study that evaluated the diagnostic accuracy and case management of clinical malaria in the primary health services of a rural area in south-eastern Tanzania provide a good example [24]. In this study, a representative sample of pediatric consultations was carefully examined at peripheral health facilities throughout Kilombero District. While the attending health workers diagnosed 640 (41.1%) of all consultations as clinical malaria cases, the study established only 397 (25.5%) of all consultations to be malaria cases by using a blood slide [24]. Furthermore, 118 (30.2%) of confirmed malaria cases that were misdiagnosed as other infections by the attending clinicians went home without antimalarial drug prescription. Likewise, some children who were misdiagnosed as malaria cases by clinicians went home with only an antimalarial which obviously was not going to help. In another study conducted in rural areas of Morogoro and Dodoma regions in Tanzania, on average only 22% of the assessment tasks required by the IMCI guidelines were performed by the attending clinicians [25].

The reported shortage of drugs and health workers is not a new finding [7,26,27]. According to the MoH Sector Strategic Plan (2003), the majority of skilled health workers in Tanzania work in large cities, and as a result, rural facilities are left understaffed [27]. Caretakers experiences related to drugs and health workers shortage in this study were disturbing. The reported practice regarding distributing one syrup among several children is unacceptable. This practice may not only lead to severe disease and death of insufficiently treated children but also development of drug resistance. In Tanzania, low cadre nurses working at PHC facilities are trained to work under instructions from clinical officers or medical doctors. Caretakers in this study reported the nurses to be the ones making the diagnoses and prescribing drugs at some of the PHC facilities when the clinicians were not present. This practice could result in mismanagement of children as the attending nurses may not have sufficient knowledge to make the correct diagnoses and give correct treatment. In a study mentioned above on misdiagnosis of malaria, 121 (7.9%) and 71 (4.6%) of consultations were made by the untrained nurses and trained nurses respectively [24].

### Accessibility and affordability of services

Caretakers reported inaccessibility of services at night from dispensaries in their area. This does not seem to support the primary objective of PHC facilities, which is to improve access of care to poor people who can not afford to travel to distant hospitals. As opposed to day time, it is always a challenge to travel to a higher level hospital at night if the need arises, due to lack of public transport. In addition, other alternatives of care like drug shops are also closed. This leaves poor people with fewer options. Caretakers in this study expressed their concerns about this problem at the dispensary level especially in relation to the safety of the newborn babies when pregnant women go into labour at night. The reported occasional skipping of hourly injections until the next working day as a result of inaccessibility of services at night and during the weekends and holidays is another example of the dangers implied.

Children below five years are exempted from the official user fees under the cost-sharing policy of Tanzania [28,29]. However, caretakers in this study reported being denied of certain services when they could not pay. Caretakers from different villages reported paying for different services, while in one of the villages they reported not to pay at all. The issue of unofficial payments is not a new finding; it has been documented previously by other studies in Tanzania [30-33]. This indicates poor monitoring of PHC facilities.

### Acceptability of services

Health services must be respectful of medical ethics, culturally appropriate and gender sensitive [34]. Several concerns were raised by caretakers in this study regarding how health workers treated them. Concerns regarding the lack of urgency, unnecessary delays and lack of sympathy to sick children may not only lead to under-utilization of services at such facilities but could also result in unnecessary child deaths. The issues of poor provider-patient relations in Tanzania have previously been documented [30]. A study in Kenya also reported similar findings [35]. Patients in this study complained of indecent behaviour by the attending nurses at the studied government hospitals, such as discrimination, lack of respect, and impolite languages and ignorance.

Referral, which is a process by which a health worker transfers the responsibility of patient care, temporarily or permanently, to another health professional [36], is a critical part of appropriate primary care and the Integrated Management of Childhood Illness (IMCI) strategy [37]. As a rule, the referring physician should clearly specify the objectives of the referral in the referral letter. The use of verbal referral without any written documentation was another practice at PHC facilities that was complained about by caretakers in our study. Caretakers’ wish of having a written document to present to the referred hospital is valid. This document could prevent giving the same treatments at a referred hospital that might have failed at a referring facility, hence avoiding unnecessary delays in the provision of appropriate care.

Poor quality of services at PHC facilities in Tanzania, characterized by the chronic shortage of equipments, supplies and staff, has previously been documented in other studies [6,7,26,30,38]. This not only affects care-seekers but could also be demotivating for health workers working at this level of care. In a study conducted in three districts of Kilimanjaro region in northern Tanzania, it was reported that clinical officers described the lack of laboratory services as “gambling with the health of patients” [39]. They claimed that people in their communities knew what quality of services they wanted, and some community members looked for the quality they simply could not provide.

At the same time, caretakers in this study acknowledged the fact that health workers were not sufficient and that it is impossible for the same health worker to work day and night without rest. Several other studies have attributed poor health workers’ performances with poor working conditions, including low salaries [40,41]. Our study findings indicate that, the quality of care could be solved by focusing on both the individual health workers and the conditions under which they work.

### Methodological strengths and limitations

The current study was complementary to the hospital-based survey [12] and was designed to provide the community views on the quality of care offered by PHC facilities in their area. The results from this qualitative study were similar to the findings from the hospital-based quantitative survey. The fact that the two studies from the same area applying different methodology give similar conclusions adds to the validity of the findings from both studies.

The focus groups were somewhat bigger than what is the standard. This was because more than expected of the caretakers that were approached were willing to participate. However, we did not encounter any difficulties during the discussions as a result of group size. The higher number of female caretakers participating as compared to male caretakers was expected, and it can be seen as strength of the study, since women are commonly the ones taking children to health facilities in the study area.

## Conclusion

Our findings support the observations made in the World Health Report of 2008 [11], that neglecting people’s needs and expectations may contributes to making health care services less relevant to the communities they serve. Caretakers in this study have pointed out important weaknesses in services provided at the PHC level. Practices regarding partial drugs administrations, skipping of injections, unofficial payments and consultations by unskilled health care providers need urgent action. These practices hamper the quality of services at PHC facilities, previously defined in terms of availability, affordability and acceptability, and hence perpetuate the problem of bypassing this level of care. Good procedures for referrals which is crucial to a functioning PHC, were also reported to be deficient. There is also a need for proper accountability mechanisms to govern appropriate allocation and monitoring of health care resources and services in Tanzania.

## Abbreviations

MoH, Ministry of Health; MOHW, Ministry of Health and Welfare; PHC, Primary Health Care; WHO, World Health Organization.

## Competing interests

The authors declare that they have no competing interests.

## Authors’ contributions

CK planned and wrote the protocol, collected and analysed data, and drafted the manuscript. GK had substantial contribution to the protocol development as well as data analysis and writing the manuscript. SGH and KMM supervised data analysis and contributed in writing the manuscript. All authors have read and approved the final version.

## Pre-publication history

The pre-publication history for this paper can be accessed here:

http://www.biomedcentral.com/1472-6963/12/158/prepub

## References

[B1] The United Republic of Tanzania: National Health Policy Ministry of Health 2003

[B2] Reerink IH Sauerborn R Quality of primary health care in developing countries: recent experiences and future directions Int J Quality Health Care 1996 8 2 131 10.1093/intqhc/8.2.131 8792168

[B3] Baltussen R Yé Y Haddad S Sauerborn RS Perceived quality of care of primary health care services in Burkina Faso Health Policy Plan 2002 17 1 42 10.1093/heapol/17.1.42 11861585

[B4] Tanzania survey points to high risk of maternal death Safe Mother 1994 13 1 2 12345452

[B5] Kruk ME Mbaruku G McCord CW Moran M Rockers PC Galea S Bypassing primary care facilities for childbirth: a population-based study in rural Tanzania Health Policy Plan 2009 24 4 279 88 10.1093/heapol/czp011 19304785

[B6] Agyemang-Gyau P, Mori AE: The ability and willingness to pay for health care: The Case of Bumbuli area in Lushoto district. In Proceedings of the 17th Annual Scientific Conference of the TPH: 23–26 Mkonge Hotel Tanga; November 1998 1999 24 26

[B7] Gilson L Magomi M Mkangaa E The structural quality of Tanzanian primary health facilities Bull World Health Organ 1995 73 1 105 7704920 PMC2486583

[B8] Audo MO Ferguson A Njoroge PK Quality of health care and its effects in the utilisation of maternal and child health services in Kenya E Afr Med J 2006 82 11 10.4314/eamj.v82i11.9407 16463747

[B9] Akin JS Hutchinson P Health-care facility choice and the phenomenon of bypassing Health Policy Plan 1999 14 2 135 10.1093/heapol/14.2.135 10538717

[B10] Low A Coeyere D Shivute N Brandt LJ Patient referral patterns in Namibia: identification of potential to improve the efficiency of the health care system Int J Health Plan M 2001 16 3 243 257 10.1002/hpm.628 11596560

[B11] World Health Organization: Primary health care now more than ever Geneva 2008

[B12] Kahabuka C Kvåle G Moland KM Hinderaker SG Why caretakers bypass PHC facilities for child care? - Acase of rural Tanzania BMC Health Serv Res 2011 11 1 315 10.1186/1472-6963-11-315 22094076 PMC3234197

[B13] The United Republic Of Tanzania: 2002 Population and Housing Census General Report 2002

[B14] National Bureau of Statistics, Tanga Regional Commissioners office: Tanga Regional Socio-economic Profile United Republic of Tanzania 2008

[B15] Kamugisha ML Gesase S Mlwilo TD Mmbando BP Segeja MD Minja DT Massaga JJ Msangeni HA Ishengoma DR Lemnge MM Malaria specific mortality in lowlands and highlands of Muheza district, north-eastern Tanzania Tanzan Health Res Bull 2007 9 1 32 37 17547098 10.4314/thrb.v9i1.14289

[B16] National Bureau of Statistics: Tanzania Demographic and Health Survey (TDHS 2004–2005) United Republic of Tanzania 2005

[B17] Smith CP Motivation and personality: Handbook of thematic content analysis Cambridge Univ Pr 1992

[B18] Aday LA Andersen R A framework for the study of access to medical care Health Serv Res 1974 9 3 208 4436074 PMC1071804

[B19] Obrist B Iteba N Lengeler C Makemba A Mshana C Nathan R Alba S Dillip A Hetzel MW Mayumana I Schulze A Mshinda H Access to health care in contexts of livelihood insecurity: a framework for analysis and action PLoS Med 2007 4 10 1584 1588 17958467 10.1371/journal.pmed.0040308 PMC2039761

[B20] Gouws E Bryce J Habicht JP Amaral J Pariyo G Schellenberg JA Fontaine O Improving antimicrobial use among health workers in first-level facilities: results from the multi-country evaluation of the Integrated Management of Childhood Illness strategy Bull World Health Organ 2004 82 7 509 515 15508195 PMC2622903

[B21] WHO: Pocket book of hospital care for children: Guidelines for the management of common illnesses with limited resources Geneva: WHO Press 2005

[B22] United Republic of Tanzania: National Malaria Medium Term Stratergic Plan 2002–2007 Ministry of Health Malaria control series 2002

[B23] O'Meara WP Mangeni JN Steketee R Greenwood B Changes in the burden of malaria in sub-Saharan Africa Lancet Infectious Diseases 2010 10 8 545 555 10.1016/S1473-3099(10)70096-7 20637696

[B24] Font F Alonso Gonzalez M, Nathan R, Kimario J, Lwilla F, Ascaso C, Tanner M, Menendez C, Alonso P: Diagnostic accuracy and case management of clinical malaria in the primary health services of a rural area in south eastern Tanzania Trop Med Int Health 2001 6 6 423 428 10.1046/j.1365-3156.2001.00727.x 11422955

[B25] Mæstad O Torsvik G Aakvik A Overworked? On the relationship between workload and health worker performance J Health Econ 2010 29 5 686 698 10.1016/j.jhealeco.2010.05.006 20633940

[B26] SDC: Views of the poor: The perspectives of rural and urban poor as recounted through their stories and pictures SDC 2003

[B27] Ministry of Health, United Republic of Tanzania Second Health Sector Strategic Plan (July 2003-June 2006) 2003

[B28] Ministry of Health, United Republic of Tanzania National Health Policy 2003

[B29] Mubyazi GM The Tanzanian Policy on Health-care fee waivers and exemptions in practice as compared with other developing countries: Evidence from recent local studies and international literature E Afr J Public Health 2004 1 1

[B30] Mamdani M Bangser M Poor People's Experiences of Health Services in Tanzania: A Literature Review Reprod Health Matters 2004 12 24 138 53 15626204 10.1016/s0968-8080(04)24135-0

[B31] Manzi F Schellenberg JA Adam T Mshinda H Victora CG Bryce J Out-of-pocket payments for under-five health care in rural southern Tanzania Health Policy Plan 2005 20 suppl 1 i85 16306074 10.1093/heapol/czi059

[B32] Stringhini S Thomas S Bidwell P Mtui T Mwisongo A Understanding informal payments in health care: motivation of health workers in Tanzania Hum R Health 2009 7 1 53 10.1186/1478-4491-7-53 PMC2711965 19566926

[B33] Mæstad O Mwisongo A Institute CM Informal payments and the quality of health care in Tanzania: results from qualitative research Chr. Michelsen institute (CMI) 2007

[B34] Baker D AAAQ Framework. 2010. Available from http://phrtoolkits.org/toolkits/medical-professionalism/the-human-rights-basis-for-professionalism-in-health-care/aaaq-framework

[B35] Ojwang BO Ogutu EA Matu PM Nurses' impoliteness as an impediment to patients' rights in selected Kenyan hospitals Int J Health and Human Right 2010 12 2 21178193

[B36] Akande TM Referral system in Nigeria Study of a tertiary health facility 2004

[B37] World Health Organization, UNICEF: Handbook IMCI Integrated Management of Childhood Illness Geneva 2006

[B38] Abel-Smith B Rawal P Can the poor afford'free'health services? A case study of Tanzania Health Policy Plan 1992 7 4 329 10.1093/heapol/7.4.329

[B39] Manongi RN Marchant TC Bygbjerg IC Improving motivation among primary health care workers in Tanzania: a health worker perspective Hum R Health 2006 4 1 6 10.1186/1478-4491-4-6 PMC1450300 16522213

[B40] Songstad NG Rekdal OB Massay DA Blystad A Perceived unfairness in working conditions: The case of public health services in Tanzania BMC Health Serv Res 2011 11 1 34 10.1186/1472-6963-11-34 21314985 PMC3045290

[B41] Willis-Shattuck M Bidwell P Thomas S Wyness L Blaauw D Ditlopo P Motivation and retention of health workers in developing countries: a systematic review BMC Health Serv Res 2008 8 1 247 10.1186/1472-6963-8-247 19055827 PMC2612662

